# Effects of Acupuncture on the Outcomes of Assisted Reproductive Technology: An Overview of Systematic Reviews

**DOI:** 10.1155/2018/7352735

**Published:** 2018-09-20

**Authors:** Jin Xi, Hao Chen, Zhi-hang Peng, Zong-xiang Tang, Xiang Song, You-bing Xia

**Affiliations:** ^1^Nanjing University of Chinese Medicine, Nanjing, China; ^2^Department of Epidemiology & Biostatistics, Nanjing Medical University, Nanjing, China; ^3^Xuzhou Medical University, Xuzhou, China

## Abstract

**Objectives:**

To conclude the evidence from systematic reviews (SRs) and meta-analyses assessing the effectiveness of acupuncture to treat couples with subfertility undergoing ART.

**Methods:**

We searched the major databases from their inception to March 2018: PubMed, Embase, The Cochrane Library, China National Knowledge Infrastructure (CNKI), Wanfang Database, Chongqing VIP, and Sino-Med (the Chinese database). The primary outcomes of the overview were live birth and clinical pregnancy, and secondary outcomes were ongoing pregnancy, miscarriage, and adverse events. Study selection, quality assessment, and data extraction were performed independently by two review authors. Review methodological quality was assessed by using the AMSTAR tool, and the quality of the evidence was rated by GRADE methods.

**Results:**

Eleven systematic reviews were included and published between 2009 and 2017. Our study showed that the acupuncture treatment seems to be a useful tool to improve the clinical pregnancy rate in patients who undergo assisted reproduction therapy. However, there was no evidence that acupuncture had any effect on live birth rate, ongoing pregnancy rates, or miscarriage regardless of whether acupuncture was performed around the time of oocyte retrieval or around the day of embryo transfer; this evidence is inconclusive because of the low quality of the included studies.

**Conclusions:**

The evidence for acupuncture to treat couples with subfertility undergoing ART remains unclear. Further research is needed, with high-quality trials undertaken and reported.

## 1. Introduction

With rapid development of the economy, changes in lifestyles, and aggravation of environmental pollution, the incidence of infertility has gained increased worldwide attention. According to the Centers for Disease Control and Prevention's (CDC) 2011–2015 report, 12.1% of American women aged 15–44 years have impaired ability to conceive or carry a baby to term; the majority (7.3 million) seeks fertility treatment [1]. In vitro fertilization has revolutionized the treatment of infertility. For many people, it provides the last possibility for pregnancy, and it is useful in almost all causes of sterility. But these results are still modest and far from the expectations of couples since only 25% of patients deliver after an attempt and 40% to 50% after several attempts [2]. Furthermore, because of expensive procedure, only some couples can afford only a limited number of treatments. Repeated cycles bring enormous economic pressure on the patients and their families. Therefore, it is important to maximize the efficiency of the procedure. Many patients have turned to complementary and alternative medical (CAM) treatments such as acupuncture to increase the success rate of assisted reproductive technology (ART) [3].

Acupuncture is an important part of traditional Chinese medicine and traces back at least 3,000 years. Recently, it has gained significant popularity in the Western world, due to its lack of side effects, convenience, and unique effect on general well-being [4]. It relies on the placement of fine needles along specific acupoint. After placement, the needle is manipulated via manual needling, electrical stimulation (electro acupuncture), heat (moxibustion), or laser energy to achieve the “de qi” which means the arrival of vital energy. Auricular acupuncture applies auricular microsystems, based on the somatic tissue of the external auricular pavilion and principally on being directly related to the central nervous system or internal organs. All acupuncture methods have been proved to be safe [5, 6].

The increased use of acupuncture in the management of infertility has been reflected in the expansion of the number of clinical trials and systematic reviews (SRs) studying this type of treatment, but its effectiveness has remained controversial. Some studies showed that acupuncture was significant in improving ART outcomes [7]. The specific rationales were displayed as follows. First, acupuncture can increase blood flow of the uterus and reduce uterine artery blood flow impedance [8]. Second, acupuncture can improve ovulation by modulating the central and peripheral nervous systems, endocrine systems and the neuroendocrine, the ovarian blood flow, and metabolism [9, 10]. Third, the acupuncture regimen can possibly improve pregnancy rate by abating stress around embryo transfer [11, 12]. The outcomes of recent studies, however, have been adverse and have shown that the role of acupuncture in the ART process was negligible or even nonexistent [13].

Many recent meta-analyses and SRs have analyzed the effects of acupuncture among women undergoing ART, and controversial results have been reported.

The aim of this paper was to assess the effectiveness of acupuncture to treat the couples with subfertility undergoing ART by summarising the evidence from SRs and meta-analyses.

## 2. Materials and Methods

### 2.1. Criteria for Considering Reviews

#### 2.1.1. Type of Studies

We selected SRs and meta-analyses that were based on randomized controlled trials (RCT). Narrative reviews and other overviews were excluded. The language placed in any of the studies is limited to Chinese and English.

#### 2.1.2. Types of Participants

Participants in eligible studies were infertile couples undergoing ART whether primary infertility or secondary infertility, and any type of acupuncture at any or all time points before, during, or after ART with the purpose to improve the ART outcome.

#### 2.1.3. Types of Interventions

The following studies were considered: SRs or meta-analyses comparing acupuncture treatment of participants versus no treatment, placebo or sham acupuncture during controlled ovarian stimulation (COS), in vitro fertilization (IVF), intracytoplasmic sperm injection (ICSI), and frozen-thawed embryo transfer (FET).

The therapeutic intervention includes traditional needling acupuncture, electroacupuncture, and moxibustion with warming needle, auricular acupuncture, and laser acupuncture. We excluded studies that did not involve skin penetration, such as tap-pricking, scrapping, point injection, acupressure, or blood-letting puncture and cupping.

Needling in the control groups could be with either placebo acupuncture or sham needle; no needling treatment is also considered. We excluded studies comparing different acupuncture treatments alone.

#### 2.1.4. Types of Outcomes

The primary outcome of this overview was live birth rate and clinical pregnancy rate. Secondary outcomes were ongoing pregnancy, miscarriage, and side effects.

### 2.2. Search Strategy

We searched digital databases for relevant studies: PubMed (1977 to March 2018), Embase (1974 to March 2018), The Cochrane Library (March 2018), Chinese databases such as the China National Knowledge Infrastructure (CNKI) (1982 to March 2018), Wanfang Database (1990 to March 2018), Chongqing VIP (1989 to March 2018), and the Chinese database Sino-Med (previously called the Chinese Biomedical Database) (1990 to March 2018). The reference lists of relevant articles were examined to identify citations not captured by electronic searches. The corresponding authors were contacted for missing information.

The following terms were searched as free text terms and Medical Subject Headings terms: acupuncture, acupuncture therapy, electro acupuncture, acupoint, needle, warm needling, acupuncture plus moxibustion, auricular acupuncture, auricular needle, ear acupuncture, and moxibustion; reproduce, ART, assisted reproductive, assisted conception, infertility, in vitro fertilization, IVF, embryo transfer, ICSI, and intracytoplasmic sperm injection. And the Medical Subject Headings terms were “acupuncture therapy”, “reproduction”, “reproductive Techniques, Assisted” and “infertility”. We combined these searches strategy with a filter for systematic review and meta-analysis.

The following terms were searched in the Chinese database: ZHEN JIU (which means “acupuncture”), ZHEN CI(“acupuncture”), HAO ZHEN (“acupuncture”), TI ZHEN(“acupuncture”), DIAN ZHEN (“electro acupuncture”), WEN ZHEN(“warm needling”), ER ZHEN (“auricular acupuncture”), JIU (“moxibustion”) and XUE WEI (“acupoint”) and BU YUN (“infertility”), SHEN ZHI (“reproduce”), FU ZHU SHENG ZHI (“assisted reproductive”), TI WAI SHOU JING(“in vitro fertilization”), PEI TAI YI ZHI (“embryo transfer”), SHI GUAN YING ER (“in vitro fertilization”), and LUAN PAO JIANG NEI DNA JING ZI XIAN WEI ZHU SHE (“intracytoplasmic sperm injection”). (See the Appendix for details of the literature search.)

### 2.3. Date Collection and Extraction

The titles and abstracts of all searches, hiding the name of the study author, were independently read by two reviewers, and then the full texts of all potentially eligible articles were obtained. Two review authors independently examined these full text articles in accordance with the inclusion criteria and selected eligible studies for inclusion in the review. In cases of duplicate publication, the most recent and complete versions were selected. Any disagreement in the process was resolved by consensus or arbitration by an experienced and authoritative third reviewer.

Two reviewers extracted data independently using a standardized data extraction form. The following specific characteristics were extracted from each study: the first author, year of publication, number of RCTs, number of participants, details of the acupuncture intervention, type of control, and outcome measures. The corresponding authors were contacted by email for missing information.

### 2.4. Assessment of Methodological Quality of Included Reviews

Quality assessment of the reviews was performed by Assessment of Multiple Systematic Reviews (AMSTAR) criteria, a validated instrument with good construct validity and reliability [14]. It contains eleven items, scored as “Yes,” “No,” “Unclear” on a checklist. Two authors independently assessed these domains, and any disagreements were resolved by consensus or discussion with the third author.

### 2.5. Quality of Evidence from Primary Studies in Included Reviews

The Grades of Recommendations, Assessment, Development and Evaluation (GRADE) approach was used to assess the level of evidence and summarise each outcome [15]. GRADE profiler software for Windows V.3.6 (GRADE working group) was used. The following criteria were taken into account: risk of bias (that is study limitations), inconsistency of effect, indirectness, imprecision, and publication bias. Two authors independently assessed the five items and resolved any disagreements through consensus or discussions with the third author. The level of evidence was categorized into four levels: high, moderate, low, or very low.

### 2.6. Statistical Analyses

The narrative description of the included studies was provided, and the network meta-analysis was not undertaken. The Forest plots were performed with StataMP 14.0.

## 3. Results

### 3.1. Systematic Review Search and Screening Results

**Figure 1:**
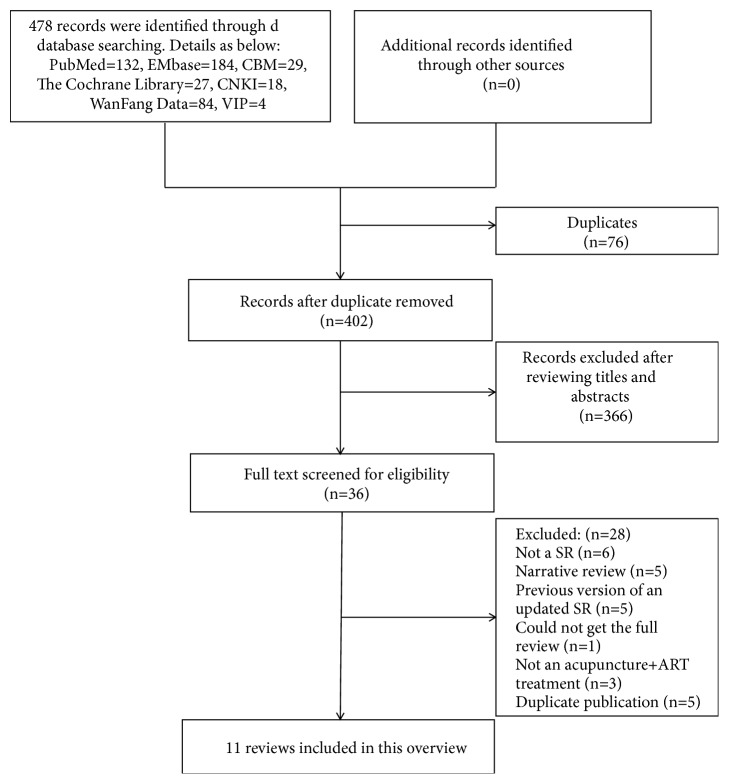
Flow diagram of systematic review selection.

A total of 478 citations were acquired from the electronic search, and 76 duplicated articles were identified and excluded. 366 citations were excluded after screening the titles and abstracts for a variety of reasons, such as types of studies, interventions, and patients. Therefore, the full texts of the remaining 36 citations were retrieved for further evaluation. Twenty-five publications were excluded for the following reasons: six [16–21] were not SRs, five [4, 10, 22–24] were narrative reviews, five [25–29] were early versions of an updated SR, one [30] could not get the full review, three [31–33] were not an acupuncture + ART treatment, and five [34–38] were duplicate publications. Thus, a total of 11 systematic reviews were finally included in this overview. The selection process is recorded with a flow chart in Figure 1.

### 3.2. Study Characteristics

The included reviews were published between 2009 and 2017; nine were published in English and three in Chinese. The number of RCTs included in the SRs varied widely, ranging from 4 to 32 studies; the quality assessment scales of the original studies varied across the included systematic reviews: ten used the risk of bias from the Method Guidelines for Systematic Reviews in the Cochrane Review Group, and one [39] adopted the modified Jadad scale. The main characteristics of the studies are showed in Table 1.

### 3.3. Methodological Quality of Included Reviews

**Figure 2:**
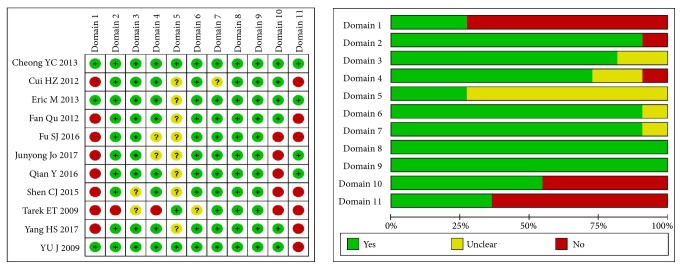
AMSTAR assessment of the included studies. Domain 1. Was an “a priori” design provided? Domain 2. Was there duplicate study selection and data extraction? Domain 3. Was a comprehensive literature search performed? Domain 4. Was the status of publication (i.e., grey literature) used as an inclusion criterion? Domain 5. Was a list of studies (included and excluded) provided? Domain 6. Were the characteristics of the included studies provided? Domain 7. Was the scientific quality of the included studies assessed and documented? Domain 8. Was the scientific quality of the included studies used appropriately in formulating conclusions? Domain 9. Were the methods used to combine the findings of studies appropriate? Domain 10. Was the likelihood of publication bias assessed? Domain 11. Was the conflict of interest included?. *∗*LEFT: summary of the AMSTAR assessments in the 11 SRs. *∗*RIGHT: graphical representation of the overall AMSTAR assessments in the eleven domains. Green, yellow, and red represent YES, UNCLEAR, and NO. Length of the rectangles (green, yellow, or red) shows the percentage of studies with YES, UNCLEAR, or NO for the eleven domains analyzed.

The quality of the included reviews was rated by AMSTAR. The number of SRs meeting the criteria for these items varied widely, and six items were satisfied: all reviews that described the characteristics of the included studies; all reviews that assessed study quality; all reviews that used the scientific quality appropriately in formulating conclusions; all reviews that combined the studies using appropriate methods; all reviews that have duplicate study selection and data extraction; ten reviews that achieved a comprehensive literature search; and eight reviews that searched the grey literature. In contrast, four items accounted for the major methodological limitations: only three reviews provided an “a priori” design; three reviews listed included and excluded studies; five reviews assessed the likelihood of publication bias; five reviews included the conflict of interest. See Figure 2 for details.

### 3.4. Quality of Evidence from Primary Studies in Included Reviews

The quality levels of evidence of all outcomes from primary studies determined by GRADE were low or very low because of the study limitations within the trials, inconsistency, and the possibility of publication bias (Table 2).

### 3.5. Outcomes

**Figure 3:**
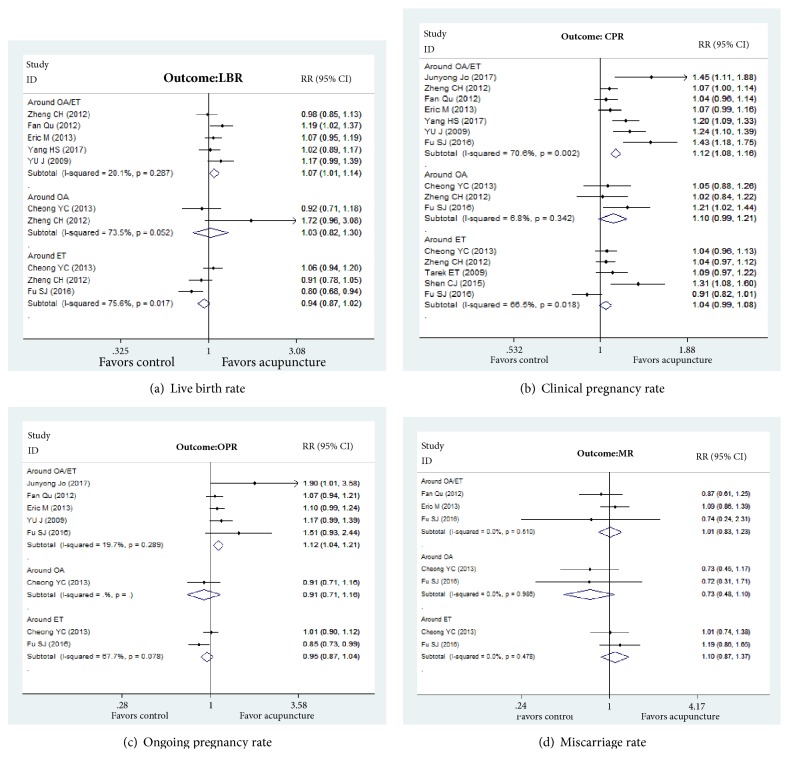
Forests plot of outcomes.

The outcome measures looked at by different studies include live birth rate, clinical pregnancy, ongoing pregnancy rate, miscarriage rate, and side effects. Due to the low incidence of these outcomes, some reviews use Odds Ratio (OR) instead of Relative Ratio (RR) to assess outcomes; we compare them with RR accounting from the raw data, with Forest plot. (See Figures 3(a), 3(b), 3(c), and 3(d) for details.)

#### 3.5.1. Live Birth Rate

Five SRs [13, 39–42] synthesized the clinical evidence about acupuncture increased live birth rates (LBR), and four [13, 39–41] showed no statistical LBR differences between all acupuncture groups and all control groups; only Yang and colleagues [42] showed that acupuncture had a slightly stronger effect in the increase of LBR. Two studies [40, 43] reported on the LBR around the time of oocyte retrieval and showed that there was no evidence of a difference between the two groups; three SRs [40, 43, 44] assessed the effectiveness of acupuncture in the improvement of LBR on the day and around embryo transfer (ET) and showed no significant differences between the acupuncture and control groups.

#### 3.5.2. Clinical Pregnancy Rate

Seven SRs [13, 39–42, 44, 45] summarised the evidence for using acupuncture to increase clinical pregnancy outcome, and four of them [39, 40, 42, 45] showed a clear difference between acupuncture groups and control groups and demonstrated that acupuncture could significantly improve the clinical pregnancy; three studies [13, 41, 44] showed no significant difference in the clinical pregnancy rate (CPR) between the acupuncture and control groups. Three studies [40, 43, 44] reported on the CPR around the time of oocyte retrieval, and two studies [40, 43] showed no significant differences between the two groups around the time of oocyte retrieval; Fu SJ [44] reported that acupuncture can improve the CPR compared with the control group. Five studies [38, 40, 43, 44, 46] provided the evidence on the effectiveness of acupuncture in improving CPR on the day and around ET. Shen CJ [38] showed that acupuncture only performed at the time of ET did not significantly improve the CPR, while showing a pooled benefit of acupuncture for CPR when performed at follicle phase and 25 min before and after ET, as well as 30 min after ET and the implantation phase. But the other studies [40, 43, 44, 46] found no favorable effects of acupuncture compared to the control groups around the time of ET.

#### 3.5.3. Ongoing Pregnancy Rate

Five SRs [13, 39, 41, 44, 45] synthesized evidence on the effectiveness of acupuncture for improving the ongoing pregnancy rate. Junyoung Jo [45] showed the ongoing pregnancy rate (OPR) was significantly increased in the acupuncture group compared with the no treatment group, and others [13, 39, 41, 44] showed no significant difference in the ongoing pregnancy outcome between the acupuncture and control groups. Cheong YC [43] demonstrated that there were no statistically significant differences whenever around ET or OA.

#### 3.5.4. Miscarriage Rate

Three SRs [13, 41, 44] provided evidence on the use of acupuncture in the decrease of miscarriage and found no significant difference in the miscarriage outcome between the acupuncture and control groups; two studies [43, 44] reported miscarriage around the time of oocyte retrieval and showed no distinct miscarriage rate (MR) differences between all acupuncture groups and all control groups. Two studies [43, 44] summarised evidence on the effectiveness of acupuncture to reduce MR on the day and around ET and showed no significant differences between acupuncture and control groups.

#### 3.5.5. Adverse Events

Of the 11 SRs, eight [38, 39, 41–44, 46, 47] did not describe any information on adverse events, and three [13, 40, 45] reported that there were no any adverse events that occurred during treatment.

## 4. Discussion

### 4.1. Summary of Evidence

This overview of eleven SRs provided the clinical evidence on the effectiveness of acupuncture to improve the outcomes of ART from 210 primary studies that included 44,619 participants. Our overview of SRs obtained the following results: (1) There was no evidence that acupuncture had any effect on live birth rate regardless of whether acupuncture was performed around the time of oocyte retrieval or around the day of embryo transfer. Maybe because the quality of the transplanted embryo is most important for the outcomes of IVF, in addition, the condition of the endometrium and the systemic health status of the woman are involved. But the acupuncture treatment that is accompanied by the assisted reproductive technology cannot fundamentally affect the condition of the ovum, which is essential for the quality of the embryo. Therefore, the impact of acupuncture on overall pregnancy and childbirth outcomes is limited. (2) Most SRs of our study demonstrated that the acupuncture could improve the clinical pregnancy rate in patients who undergo assisted reproduction therapy. But some studies showed no significant difference in the CPR between the acupuncture and control group, maybe due to the low quality of the included studies. Acupuncture treatment at or around the time of IVF may increase uterine blood flow, modulate immune function, inhabit uterine motility, and relieve anxiety, depression, and stress [7]. And these mechanisms of action of acupuncture are considered to result in improved CPR. (3) There was no evidence that acupuncture had any effect on ongoing pregnancy rates and miscarriage regardless of the treatment timing. (4) Because of the presence of methodological bias, inconsistency, and the possibility of publication bias, the quality levels of evidence of all outcomes from primary studies determined by GRADE were “low” or “very low”. (5) The improvement of acupuncture-related CPR during IVF was the most frequent study, followed by improvement in LBR, OPR, and MR. However, fewer reports of adverse reactions, such as multiple pregnancies and ovarian hyperstimulation, have been reported. For any clinical intervention, the patient's safety should be monitored strictly and reported as part of the trial procedure. Future studies should combine the reports of adverse reactions with detailed information of receiving acupuncture and any treatment-related events.

### 4.2. Prospects for Future Research

Based on the ability of acupuncture to increase the flow of Qi at needle insertion points, Chinese medicine practitioners have historically believed that acupuncture is particularly effective in increasing clinical pregnancy rates. But most reviews showed no significant improvement in outcomes of assisted reproductive technology when acupuncture was performed. The inability of our overview to provide evidence that acupuncture can be effective for female infertility may be due to the low quality of the included reviews. The lack of high-quality SRs may be due to small study populations, poorly designed RCTs, and methodological flaws. Further well-designed and sufficiently powered randomized trials to evaluate the effectiveness of acupuncture on ART outcomes should be carried out. In addition, more comprehensive outcomes should be observed, especially the live birth rate.

Embryo quality is one of the most important prognostic factors in IVF and oocyte quality plays a major role in the development potential of embryo [48]. Li J et al. [49] found electroacupuncture can improve the quality of oocytes and clinical pregnancy by modulating the function of integer and ovarian local circumstance of the patients who are undergoing IVF. It is believed that there is a need to study the effects of acupuncture treatment only when high-quality embryos are transplanted. Therefore, in the future the experiments on whether acupuncture can improve oocytes quality should pay more attention to.

### 4.3. Advantages and Limitations

Our study is the first summary of the effects of acupuncture on the outcomes of ART with an overview. In order to enable this overview informative for clinicians and researchers, we established strict inclusion criteria and the data were separated according to the time of treatment; however, limited to language, the study included only systematic reviews published in Chinese and English. At the same time, only the published systematic reviews were searched and may miss the unpublished literature, so there is a certain publication bias.

## 5. Conclusion

To conclude, the evidence for acupuncture to increase the success rate of ART is unclear. Thus, physicians should apply the evidence to make decisions about acupuncture for infertile women under ART with caution in clinical practice and consider the actual situation, combined with the patient's value preferences and economic factors.

## Figures and Tables

**Table 1:** Characteristics of the included studies.

First author and publication year	Publication language	No. of RCTs(No. of patients) included	Quality assessment scales of the original studies	Interventions		Outcomes
				Nature of acupuncture	Nature of control interventions	
Cheong YC, 2013	English	20(4544)	Risk of bias	EA∖MA∖AA	no treatment, placebo or sham acupuncture	(1),(2),(3),(4),(5),
Junyong Jo, 2017	English	4(430)	Risk of bias	EA∖MA	no treatment	(1),(2),(3),(5)
Zheng CH, 2012	English	24(5807)	Risk of bias	MA∖EA∖LA	no treatment, placebo or sham acupuncture	(1),(2),(3),(4),(5),
Fan Qu, 2012	English	17(3744)	Risk of bias (Cochrane Review Groups 2006)	MA∖EA∖LA∖AA	no treatment, placebo or sham acupuncture	(1),(2),(3),(4)
Eric M, 2013	English	16(4038)	Risk of bias(Cochrane Collaboration 2011)	MA	sham acupuncture, no treatment	(1),(2),(3),
Tarek ET, 2009	English	14(2870)	Risk of bias	MA∖EA∖LA	sham acupuncture, no treatment	(1)
Shen CJ, 2015	English	21(5428)	Risk of bias	MA∖EA∖LA	no treatment, placebo or sham acupuncture	(1)
Qian Y, 2017	English	30(6344)	Risk of bias	MA∖EA∖LA∖AA	no treatment, placebo or sham acupuncture	(1),(2),(3)
Yang HS, 2017	Chinese	32(4815)	Risk of bias	MA∖EA∖WN	no treatment, placebo or sham acupuncture	(1),(3)
YU J, 2009	Chinese	10(2046)	Modified Jadad scale	MA∖EA∖AA	no treatment, placebo or sham acupuncture	(1),(2),(3)
Fu SJ, 2016	Chinese	22(4553)	Risk of bias	MA∖EA∖AA∖WN	no treatment, placebo or sham acupuncture	(1),(2),(3),(4)

(1) Clinical Pregnancy Rate; (2) Ongoing Pregnancy Rate; (3) Live Birth Rate; (4) Miscarriage Rate; (5) Side Effects

MA: manual acupuncture; EA: electroacupuncture; LA: laser acupuncture; AA: auricular acupuncture; WN: warm needling

**Table:** (a) Acupuncture around the time of oocyte retrieval or ET versus control (sham, placebo, no acupuncture)

Study	Outcomes	Number of participants(studies)	Risk of bias	Inconsistency	Indirectness	Imprecision	Publication-bias	Relative effect (95% CI)	Quality of the evidence (GRADE)
Junyong Jo 2017	LBR	66(1 RCT)	not serious	not serious	not serious	serious	serious	RR 1.61(0.73,3.58)	LOW
	CPR	430(4 RCTs)	serious	not serious	not serious	serious	not serious	RR 1.35(1.05,1.74)	LOW
	OPR	164(2 RCTs)	not serious	not serious	not serious	serious	serious	RR 1.99(1.06,3.74)	LOW

Zheng CH 2012	LBR	1863(6 RCTs)	serious	serious	not serious	not serious	not serious	OR 1.09(0.74,1.60)	LOW
	CPR	5599(23 RCTs)	serious	serious	not serious	not serious	not serious	OR 1.22(1.01,1.47)	LOW

Fan Qu 2012	LBR	1990(6 RCTs)	serious	very serious	not serious	not serious	not serious	RR 1.42(0.92,2.20)	VERY LOW
	CPR	3713(17 RCTs)	serious	serious	not serious	not serious	not serious	RR 1.09(0.94,1.26)	LOW
	OPR	2392(8 RCTs)	serious	serious	not serious	not serious	not serious	RR 1.20(0.93,1.56)	LOW
	MR	373(5 RCTs)	serious	not serious	not serious	serious	not serious	RR 0.95(0.68,1.35)	LOW

Eric M 2013	LBP	3075(12 RCTs)	serious	serious	not serious	not serious	not serious	RR 1.14(0.92,1.42)	LOW
	CPR	4021(16 RCTs)	serious	serious	not serious	not serious	not serious	RR 1.12(0.96,1.31)	LOW
	OPC	3005(11 RCTs)	serious	serious	not serious	not serious	not serious	RR 1.22(0.98,1.52)	LOW
	MR	1070(12 RCTs)	serious	not serious	not serious	not serious	not serious	RR 1.09(0.85,1.40)	MODERATE

Qian Y 2017	LBR	2331(9 RCTs)	serious	very serious	not serious	not serious	not serious	OR 1.17(0.80,1.72)	VERY LOW
	CPR	6344(30 RCTs)	serious	serious	not serious	not serious	not serious	OR 1.26(1.06,1.50)	LOW
	OPR	3602(10 RCTs)	serious	serious	not serious	not serious	not serious	OR 1.14(0.87,1.48)	LOW

Yang HS 2017	LBR	1951(8 RCTs)	serious	serious	not serious	not serious	not serious	RR 1.18(0.89,1.58)	LOW
	CPR	2773(17 RCTs)	serious	serious	not serious	not serious	not serious	RR 1.43(1.15,1.77)	LOW

Yu J 2009	LBR	1333(6 RCTs)	serious	very serious	not serious	not serious	not serious	RR 1.28(0.91,1.79)	VERY LOW
	CPR	2103(11 RCTs)	serious	serious	not serious	not serious	serious	RR 1.34(1.09,1.66)	VERY LOW
	OPR	1333(6 RCTs)	serious	very serious	not serious	not serious	not serious	RR 1.28(0.91,1.79)	VERY LOW

**Table:** (b) Acupuncture before oocyte retrieval and∖or on the time of oocyte retrieval versus control (sham, placebo, no acupuncture)

Study	Outcomes	Number of participants(studies)	Risk of bias	Inconsistency	Indirectness	Imprecision	Publication bias	Relative effect (95% CI)	Quality of the evidence (GRADE)
Cheong YC 2013	LBR	464(2 RCTs)	serious	not serious	not serious	not serious	serious	OR 0.87(0.59,1.29)	LOW
	OPR	464(2 RCTs)	serious	not serious	not serious	not serious	serious	OR 0.86(0.58,1.26)	LOW
	CPR	912(6 RCTs)	serious	serious	not serious	not serious	not serious	OR 1.12(0.78,1.62)	LOW
	MR	262(4 RCTs)	serious	not serious	not serious	serious	not serious	OR 0.79(0.42,1.47)	LOW

Zheng CH 2012	LBP	142(1 RCT)	serious	not serious	not serious	serious	serious	OR 2.08(0.96,4.50)	VERY LOW
	CPR	699(4 RCTs)	serious	serious	not serious	not serious	not serious	OR 1.12(0.82,1.52)	LOW

Qian Y 2017	CPR	876(5 RCTs)	serious	serious	not serious	not serious	not serious	OR 1.07(0.81,1.41)	LOW

FU SJ 2016	CPR	835(7 RCTs)	serious	not serious	not serious	not serious	serious	OR 1.40(1.05,1.86)	LOW
	MR	231(4 RCTs)	serious	not serious	not serious	serious	not serious	OR 0.69(0.26,1.86)	LOW

**Table:** (c) Acupuncture on and around the day of ET versus control (sham, placebo, no acupuncture)

Study	Outcomes	Number of participants(studies)	Risk of bias	Inconsistency	Indirectness	Imprecision	Publication bias	Relative effect (95% CI)	Quality of the evidence (GRADE)
Cheong YC 2013	LBR	2505(8 RCTs)	serious	serious	not serious	not serious	not serious	OR 1.22(0.87,1.70)	LOW
	OPR	2807(10 RCTs)	serious	serious	not serious	not serious	not serious	OR 1.10(0.80,1.52)	LOW
	CPR	3632(14 RCTs)	serious	not serious	not serious	not serious	serious	OR 1.11(0.87,1.42)	LOW
	MR	616(6 RCTs)	serious	not serious	not serious	not serious	serious	OR 1.10(0.73,1.67)	LOW

Zheng CH 2012	LBP	1647(4 RCTs)	serious	serious	not serious	not serious	not serious	OR 0.87(0.70,1.07)	LOW
	CPR	4418(14 RCTs)	serious	serious	not serious	not serious	not serious	OR 1.12(0.89,1.42)	LOW

Tarek ET 2009	CPR	1993(9 RCTs)	serious	very serious	not serious	not serious	not serious	RR 1.16(0.92,1.48)	VERY LOW

Shen CJ 2015	CPR	859(4 RCTs)	serious	very serious	not serious	not serious	serious	RR 1.39(0.90,2.13)	VERY LOW

Qian Y 2017	CPR	4513(16 RCTs)	serious	serious	not serious	not serious	not serious	OR 1.19(0.95,1.50)	LOW

FU SJ 2016	LBR	1756(7 RCTs)	serious	very serious	not serious	not serious	not serious	RR 1.06(0.79,1.42)	VERY LOW
	CPR	2668(10 RCTs)	serious	very serious	not serious	not serious	not serious	RR 1.07(0.86,1.33)	VERY LOW
	OPR	1729(5 RCTs)	serious	serious	not serious	not serious	serious	RR 1.05(0.69,1.69)	VERY LOW
	MR	772(6 RCTs)	serious	not serious	not serious	not serious	serious	RR 1.15(0.83,1.59)	LOW

## Appendix

### Details of the Literature Search Strategy

*(1) PubMed 1977 to March 2018*

  #1 acupuncture [Title/Abstract]
  #2 electro acupuncture [Title/Abstract]
  #3 acupoint [Title/Abstract]
  #4 needle [Title/Abstract]
  #5 moxibustion [Title/Abstract]
  #6 #1 or #2 or #3 or #4 or #5
  #7 acupuncture plus moxibustion [Title/Abstract]
  #8 warm needling [Title/Abstract]
  #9 auricular acupuncture [Title/Abstract]
  #10 auricular needle [Title/Abstract]
  #11 ear acupuncture [Title/Abstract]
  #12 #7 or #8 or #9 or #10 or #11
  #13 #6 or #12
  #14 reproduce [Title/Abstract]
  #15 ART [Title/Abstract]
  #16 assisted reproductive [Title/Abstract]
  #17 assisted conception [Title/Abstract]
  #18 infertility [Title/Abstract]
  #19 #14 or #15 or#16 or #17 or #18
  #20 in vitro fertilization [Title/Abstract]
  #21 IVF [Title/Abstract]
  #22 embryo transfer [Title/Abstract]
  #23 ICSI [Title/Abstract]
  #24 intracytoplasmic sperm injection [Title/Abstract]
  #25 #20 or #21 or #22 or #23 or #24
  #26 #19 or #25
  #27 #13 and #26
  #28 acupuncture therapy [MeSH Terms]
  #29 Reproduction [MeSH Terms]
  #30 Reproductive Techniques, Assisted [MeSH Terms]
  #31 infertility [MeSH Terms]
  #32 #29 or #30 or #31
  #33 #28 and #32
  #34 #27 or #33 Filters: Meta-Analysis; Systematic Reviews

*(2) Embase from 1974 to March 2018*

  #1 acupuncture.tw.
  #2 electro acupuncture.tw.
  #3 acupoint.tw.
  #4 needle.tw.
  #5 moxibustion.tw.
  #6 #1 or #2 or #3 or #4 or #5
  #7 acupuncture plus moxibustion.tw.
  #8 warm needling.tw.
  #9 auricular acupuncture.tw.
  #10 auricular needle.tw.
  #11 ear acupuncture.tw.
  #12 #7 or #8 or #9 or #10 or #11
  #13 #6 or #12
  #14 reproduce.tw.
  #15 ART.tw.#16 assisted reproductive.tw.
  #17 assisted conception.tw.
  #18 infertility.tw.
  #19 #14 or #15 or#16 or #17 or #18
  #20 in vitro fertilization.tw.
  #21 IVF.tw.
  #22 embryo transfer.tw.
  #23 ICSI.tw.
  #24 intracytoplasmic sperm injection.tw.
  #25 #20 or #21 or #22 or #23 or #24
  #26 #19 or #25
  #27 #13 and #26
  #28 exp acupuncture therapy/
  #29 exp Reproduction/
  #30 exp Reproductive Techniques,Assisted/
  #31 exp infertility/
  #32 #29 or #30 or #31
  #33 #28 and #32
  #34 #27 or #33 Limit to: Meta-Analysis; Systematic Reviews

*(3) The Cochrane Library (March 2018)*

  #1 acupuncture:ti,ab,kw or acupoint:ti,ab,kw or needle:ti,ab,kw (Word variations have been searched)
  #2 electroacupuncture:ti,ab,kw or warm needling:ti,ab,kw or acupuncture plus moxibustion:ti,ab,kw (Word variations have been searched)
  #3 auricular acupuncture:ti,ab,kw or auricular needle:ti,ab,kw or ear acupuncture:ti,ab,kw or moxibustion:ti,ab,kw (Word variations have been searched)
  #4 #1 or #2 or #3
  #5 reproduce:ti,ab,kw or ART:ti,ab,kw or assisted reproductive:ti,ab,kw or assisted conception:ti,ab,kw or (Word variations have been searched)
  #6 infertility:ti,ab,kw or in vitro fertilization:ti,ab,kw or IVF:ti,ab,kw or embryo:ti,ab,kw or embryo transfer:ti,ab,kw (Word variations have been searched)
  #7 ICSI:ti,ab,kw or intracytoplasmic sperm injection:ti,ab,kw (Word variations have been searched)
  #8 #5 or #6 or #7
  #9 #4 and #8
  #10 MeSH descriptor: [Acupuncture Therapy] explode all trees
  #11 MeSH descriptor: [Reproduction] explode all trees
  #12 MeSH descriptor: [Reproductive Techniques, Assisted] explode all trees
  #13 MeSH descriptor: [Infertility] explode all trees
  #14 #11 or #12 or #13
  #15 #10 and #14
  #16 #8 or #15

*(4) China National Knowledge Infrastructure (CNKI) from 1982 to March 2018. *SU=acupuncture(ZHENCI) + acupuncture and moxibustion(ZHENJIU) + body acupuncture(TIZHEN) + filiform needle(HAOZHEN) + electro acupuncture (DIANZHEN) + warm acupuncture(WENZHEN) + auricular acupuncture (ERZHEN) + acupoint(XUEWEI) + moxibustion(JIU) AND SU=infertility(BUYUN) + sterility(BUYU) + reproduction(SHENGZHI) + assisted reproduction (FUZHUSHENGZHI) + artificial insemination(RENGONGSHOUJING) + IVF-ET(TIWAISHOUJING-PEITAIYIZHI) + embryo(PEITAI) AND SU= systematic Reviews (XITONGPINGJIA) + meta-Analysis (HUICUIFENXI)

*(5) Chongqing VIP Database from 1989 to March 2018. *(M= acupuncture(ZHENCI) OR M= acupuncture and moxibustion(ZHENJIU) OR M= filiform needle(HAOZHEN) OR M= body acupuncture(TIZHEN) OR M= electro acupuncture (DIANZHEN) OR M= warm acupuncture(WENZHEN) OR M= auricular acupuncture (ERZHEN) OR M= acupoint(XUEWEI) OR M= moxibustion(JIU)) AND (M= infertility(BUYUN) OR M= sterility(BUYU) OR M= reproduction(SHENGZHI) OR M= assisted reproduction (FUZHUSHENGZHI) OR M= artificial insemination OR M= embryo(PEITAI) OR M= IVF-ET(TIWAISHOUJING-PEITAIYIZHI)) AND (M= systematic Reviews (XITONGPINGJIA) OR M= meta-Analysis (HUICUIFENXI))

*(6) Wanfang Database from 1990 to March 2018. *SU=acupuncture(ZHENCI) + acupuncture and moxibustion(ZHENJIU) + body acupuncture(TIZHEN) + filiform needle(HAOZHEN) + electro acupuncture (DIANZHEN) + warm acupuncture(WENZHEN) + auricular acupuncture (ERZHEN) + acupoint(XUEWEI) + moxibustion(JIU) AND SU=infertility(BUYUN) + sterility(BUYU) + reproduction(SHENGZHI) + assisted reproduction (FUZHUSHENGZHI) + artificial insemination(RENGONGSHOUJING) + IVF-ET(TIWAISHOUJING-PEITAIYIZHI) + embryo(PEITAI) AND SU= systematic Reviews (XITONGPINGJIA) + meta-Analysis (HUICUIFENXI)

*(7) The Chinese Biomedical Database from 1990 to March 2018*

  #1 acupuncture (ZHENCI).tw.
  #2 acupuncture and moxibustion (ZHENJIU).tw.
  #3 body acupuncture (TIZHEN).tw.
  #4 filiform needle (HAOZHEN).tw.
  #5 electro acupuncture (DIANZHEN).tw.
  #6 warm acupuncture (WENZHEN).tw.
  #7 moxibustion (JIU).tw.
  #8 #1 or #2 or #3 or #4 or #5 or #6 or #7
  #9 infertility (BUYUN).tw.
  #10 sterility (BUYU).tw.
  #11 reproduction (SHENGZHI).tw.
  #12 assisted reproduction (FUZHUSHENGZHI).tw.
  #13 artificial insemination (RENGONGSHOUJING).tw.
  #14 artificial insemination (RENGONGSHOUJING).tw.
  #15 IVF-ET (TIWAISHOUJING-PEITAIYIZHI).tw.
  #16 embryo (PEITAI).tw.
  #17 #9 or #10 or #11 or #12 or #13 or #14 or #15 or #16
  #18 systematic Reviews (XITONGPINGJIA).tw.
  #19 meta-Analysis (HUICUIFENXI).tw.
  #20 #18 or #19
  #21 #8 and #17 and #20
  #22 exp acupuncture therapy(ZHENJIULIAOFA)
  #23 exp Reproductive Techniques, Assisted(SHENGZHIJISHU.FUZHU)
  #24 exp meta-Analysis (HUICUIFENXI)
  #25 #22 and #23 and #24
  #26 #21 and #25
